# Identification of Breast Cancer Using Integrated Information from MRI and Mammography

**DOI:** 10.1371/journal.pone.0128404

**Published:** 2015-06-09

**Authors:** Shih-Neng Yang, Fang-Jing Li, Yen-Hsiu Liao, Yueh-Sheng Chen, Wu-Chung Shen, Tzung-Chi Huang

**Affiliations:** 1 Department of Biomedical Imaging and Radiological Science, China Medical University, Taichung, Taiwan; 2 Department of Radiation Oncology, China Medical University Hospital, Taichung, Taiwan; 3 Department of Radiology, China Medical University Hospital, Taichung, Taiwan; 4 Department of Biomedical Informatics, Asia University, Taichung, Taiwan; University of Rome, School of Medicine and Psycology, ITALY

## Abstract

**Objectives:**

Integration of information from corresponding regions between the breast MRI and an X-ray mammogram could benefit the detection of breast cancer in clinical diagnosis. We aimed to provide a framework of registration from breast MRI to mammography and to evaluate the diagnosis using the combined information.

**Materials and Methods:**

43 patients with 46 lesions underwent both MRI and mammography scans, and the interval between the two examinations was around one month. The distribution of malignant to benign lesions was 31/46 based on histological results. Maximum intensity projection and thin-plate spline methods were applied for image registration for MRI to mammography. The diagnosis using integrated information was evaluated using results of histology as the reference. The assessment of annotations and statistical analysis were performed by the two radiologists.

**Results:**

For the cranio-caudal view, the mean post-registration error between MRI and mammography was 2.2±1.9 mm. For the medio-lateral oblique view, the proposed approach performed even better with a mean error of 3.0±2.4 mm. In the diagnosis using MRI assessment with information of mammography, the sensitivity was 91.9±2.3% (29/31, 28/31), specificity 70.0±4.7% (11/15, 10/15), accuracy 84.8±3.1% (40/46, 38/46), positive predictive value 86.4±2.1% (29/33, 28/33) and negative predictive value 80.8±5.4% (11/13, 10/13).

**Conclusion:**

MRI with the aid of mammography shows potential improvements of sensitivity, specificity, accuracy, PPV and NPV in clinical breast cancer diagnosis compared to the use of MRI alone.

## Introduction

Breast cancer is the most common cancer among women. Mammography and MRI are widely utilized as noninvasive diagnostic imaging in clinical practice for evaluation of breast lesions. Digital mammography is considered the standard for radiographic imaging of the breasts with respect to both screening and clinical indications [1]. Standardized reporting using the Breast Imaging Reporting And Data Systems (BI-RADS) lexicon, developed by the American College of Radiology has been found useful as a practical way of communication between radiologists and clinicians compared to the free style, according to the opinions of radiologists [2]. Although mammography is a reasonably sensitive test for screening postmenopausal women, it is less sensitive in younger women and those with a genetic predisposition to breast cancer. This has been attributed to increased mammographic density in premenopausal women which can obscure the radiological features of early breast cancer and the faster growth of breast cancers in these populations [3]. On the other hand, breast MRI not only provides 3D information but also consistently demonstrates the higher sensitivity for detecting breast cancer than mammography including non-palpable lesion [4–6]. Clinically, breast MRI imaging provides the morphologic info (shape, contours, distribution of non-masses…) used in daily practice to categorize lesions. With aids of contrast, the levels of background parenchymal enhancement and the amount of fibroglandular tissue (FGT) shown on MRI are features of normal breast tissue. Breast MRI depicted FGT is distinguished from fat on the basis of differences in signal intensity and breast MRI is more accurate in assessment of the amount of FGT than assessment by mammography because of three-dimensional of breast information in contrast to 2D in mammography [7]. Furthermore, the shape of the time-intensity curves (kinetic curves) derived from the dynamic contrast enhanced MR images, based on the wash-in and washout patterns, can be categorized as persistently enhancing (type 1), plateau (type 2), or washout (type 3). This kinetic curve is a commonly used the dynamic contrast enhanced parameter by which to characterize a lesion, with the washout shape (type 3) carrying a high positive predictive value for breast cancer [8–9]. However, the high sensitivity for breast cancer with contrast-enhanced breast MRI was verified in numerous other studies, but a low specificity was reported in some of these studies [10–15]. Although the use of diffusion-weighted imaging is one approach that would improve specificity from lesion characterization in MRI, the new pulse sequences may not best suit the general clinical patients [16–17]. The combination of the mammography and MRI to provide further diagnostic information for the breast cancer diagnosis and aids to the surgeons in accurately localizing the lesion for advanced biopsy are highly expected in clinical radiology [18].

The fact that women are lying prone in the MR scanner with their breasts pendulous, while during X-ray mammography acquisitions women are standing with their breast pressed causes the challenge on identifying corresponding regions between X-ray mammography and MRI. In addition, while the mammograms show only a two-dimensional projection of the compressed breast including of Cranio-Caudal (CC) and Medio-Lateral Oblique (MLO) view of images, MRI provides three-dimensional images by acquiring multiple slice images of the breast freely hanging in prone position. Therefore, image registration is necessary to make structures correspondence between mammography and MRI. Several techniques have been investigated to register the mammography and MRI previously. A 3D affine transformation with twelve parameters for registration was reported by Mertzanidou et al [19]. However, geometric nonlinearities caused from mammographic compression were not taken into account. The simulations using finite element analysis (FEA) with different optimizations, including of biomechanical model and image intensity, to deform breast MRI into X-ray mammograms was reported in previous studies [20–22]. The complicated input parameters, for examples friction coefficient, boundary condition etc., and the simulations without consideration of X-ray attenuation from mammography would limit FEA application in clinical practice.

Thin-plate spline (TPS) is a commonly used deformable registration method from one image to another by selection of matching points, which is applied for global and local alignments. In this study, we propose an effective method incorporated with maximum intensity projection (MIP) and TPS method to register MRI and mammography. Furthermore, we evaluate the diagnosis of breast cancer based on the integrated information from MRI and mammography with clinical patients’ data, taking histological results as the reference. The objective in the present study is to provide a framework of registration from breast MRI to mammography and to evaluate the diagnosis using combined information from MRI and mammography.

## Materials and Methods

### Patients

Patients who underwent X-ray mammography screening and MRI as a second-level examination in our hospital from May 2013 through June 2014 were included in the study group. MRI usually was performed after mammography only in specific cases, such as dense breasts, young patients, lobular histology. The interval between the two examinations was less than one month. Forty-six lesions from forty-three patients (mean age 45 range: 30–65 years) with results of histology confirmation were retrospectively selected in this study. The demographic characteristics of the study population are summarized in Table 1. All patient identifiers were removed from the images for the study and the data collection and analysis performed in this study were approved by the Institutional Review Board of China Medical University Hospital (CMUH103-REC3-052). All breast MR imaging and mammography findings were reported according to the level of suspicion of malignancy by using the kinetic curve and the American College of Radiology Breast Imaging Reporting and Data System lexicon, 2003 [23].

**Table 1:** The findings based on mammography, MRI and histological results for our patient cohort.

BI-RADS of Mammography	BI-RADS 0		1
	BI-RADS 1		1
	BI-RADS 2		0
	BI-RADS 3		9
	BI-RADS 4A		10
	BI-RADS 4B		8
	BI-RADS 4C		3
	BI-RADS 5		11
	BI-RADS 6		0
Kinetic curve of MRI	Persistently enhancing (type 1)		14
	Plateau (type 2)		8
	Washout (type 3)		24
Histological results	Malignancy	IDC	25
		DCIS	5
		Mucinous Carcinoma	1
	Benign		15
Lesion size	>10mm^2^		25
	5-10mm^2^		17
	<5mm^2^		4
Lesion side	Left	Subareolar area	4
		upper outer quadrant (UOQ)	13
		upper inner quadrant (UIQ)	8
		lower outer quadrant (LOQ)	0
		lower inner quadrant (LIQ)	4
	Right	Subareolar area	5
		upper outer quadrant (UOQ)	7
		upper inner quadrant (UIQ)	4
		lower outer quadrant (LOQ)	1
		lower inner quadrant (LIQ)	0

### Mammography

The conventional two-view mammograms, craniocaudal and mediolateral oblique views, were obtained by clinical full-field digital mammography unit, which used molybdenum/rhodium dual tube track (GE medical systems, Senographe Essential). The mammography data were reconstructed using a 1914 × 2294 matrix with 0.09mm x 0.09 mm resolution.

### MRI

All breast MRI screening examinations were performed at 3.0T (GE medical systems, Signa HDxt) with the patient prone and by using a dedicated surface breast coil. The standard imaging protocol included a localizing MR sequence followed by a T2-weighted fat-suppressed sequence, a T1-weighted non–fat-suppressed sequence, and a bilateral T1-weighted simultaneous fat-suppressed sequence performed before and eight times after a rapid bolus injection. Contrast media (Gadodiamide, GE Healthcare, Ireland) was administered immediately after the end of first (pre-contrast) sequence as a bolus intravenous injection at a dose of 0.1 mmol/kg and at the rate of 2.6 ml/s. The high resolution MRI data were reconstructed using a 1024 × 1024 matrix and a 1.8mm slice thickness under TR = 10.35 ms, TE = 4.25 ms, FOV = 360×360 mm^2^.

### Histological diagnosis

All patients received echo-guided biopsy or MRI-guided biopsy. All samples were embedded in paraffin and serially cut for immunohistochemical stains. Histological diagnosis was determined by the pathologist.

### Image registration

**Fig 1:**
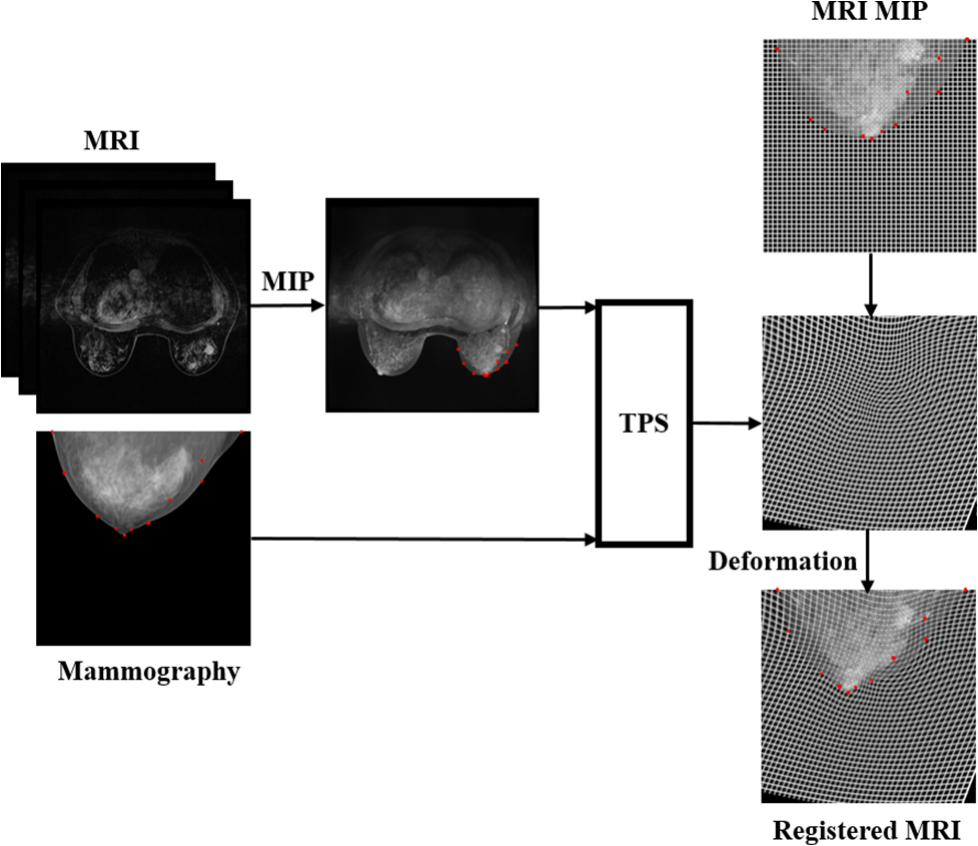
The proposed framework for MRI to mammography registration.

A projection image based on maximum intensity projection method through original MRI is the first step. TPS, a deformable image registration method, was then applied to register the MIP to mammography including both images of CC and MLO views. TPS provides the mapping function by the deformation generated between the two sets of corresponding control points on the two corresponding images [24]. Consequently, the deformed image was registered to the corresponding image in space domain. In this study, the deformation for MIP image to mammography, *f*(*x*,*y*), was defined as:

$$f\left(x,y\right)=a_{1}+a_{2}x+a_{3}y+\sum_{i=1}^{n}w_{i}U\left(\left|P_{i}-(x,y)\right|\right)$$ (1)

where x, y are the coordinates of the images, a_1_, a_2_, a_3_ determined by best matches of all control points, *w*_*i*_ is a set of mapping coefficients, |*P*_*i*_ – (*x*,*y*)| is the distance between the control point *P*_*i*_ and coordinate (x, y), U defined by *U*(*r*) = *r* ^2^ log *r* ^2^. The scheme of the proposed image registration was illustrated in Fig 1.

### Lesion analysis

MRI annotations, based on the full dynamic contrast sequence, were performed using a sphere center around the lesion while X-ray annotations were delineated using a free-form shape. The delineation in both MRI and mammography was conducted by the same radiologist. For each registration, the error was calculated as the 2D Euclidean distance between the center of mass of the annotation position in the X-ray mammogram and the center of mass of the MRI annotation projected into 2D at the final registration position. Both CC and MLO views of X-ray mammography were investigated in the analysis. The differences were expressed as the mean ± the standard deviation.

**Fig 2:**
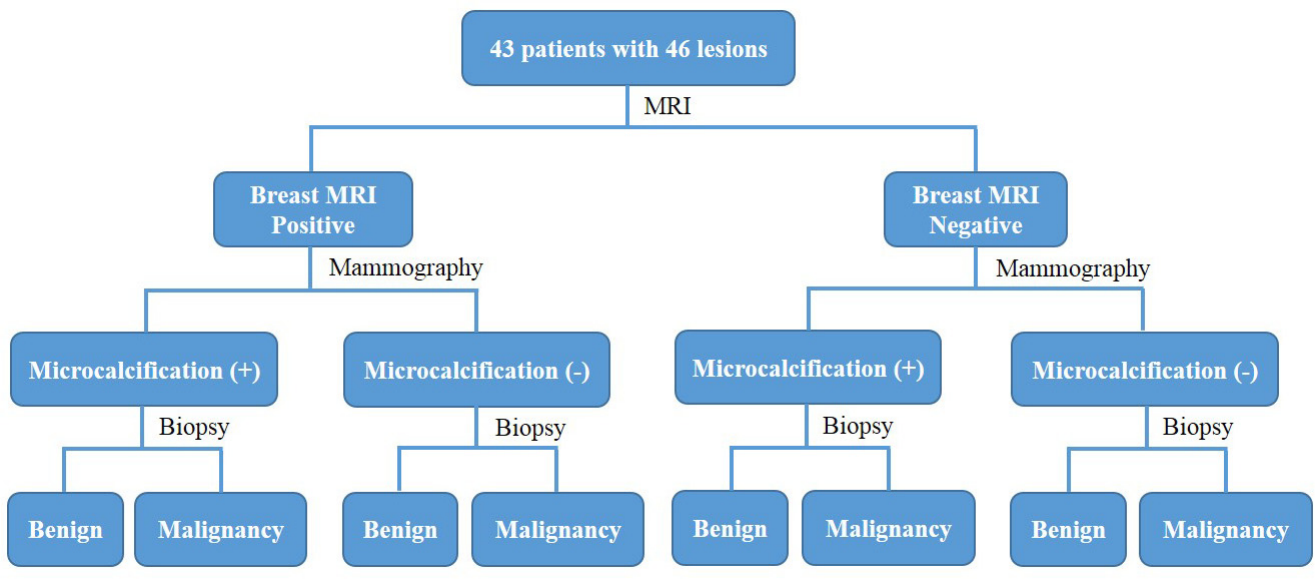
The study flowchart of evaluation of lesion using integrated information from MRI and mammography.

The lesions detected by MRI were divided to two groups as the positive and negative, based on the morphologic data (Table 2) and kinetic curve initially. The lesions indicated in irregular, lobulated, spiculated, irregular, segmental enhancement, ductal enhancement based on morphologic finding, or the type 2/type 3 in kinetic curve were in positive group and others were in negative [25]. With registration performed, the MRI annotation was deformed into the corresponding contour on mammography. Two radiologists evaluated whether the microcalcifications existed. The diagnosis using the integrated information from the registered MRI and mammography was then evaluated. The study flowchart was illustrated in Fig 2. Sensitivity, specificity, accuracy, positive predictive value (PPV) and negative predictive value (NPV) of the diagnosis were evaluated in this study. The statistical analysis was performed using software SPSS and the kappa statistic was used to assess the variability of microcalcification evaluation by the two radiologists.

**Table 2:** MRI morphologic data in our patient cohort.

Mass	Shape	Oval	8
		Irregular	18
		Lobulated	10
		Round	1
	Margin	Smooth	11
		Spiculated	11
		Irregular	15
Non-mass	Distribution	Segmental enhancement	8
		Ductal enhancement	1

## Results

**Fig 3:**
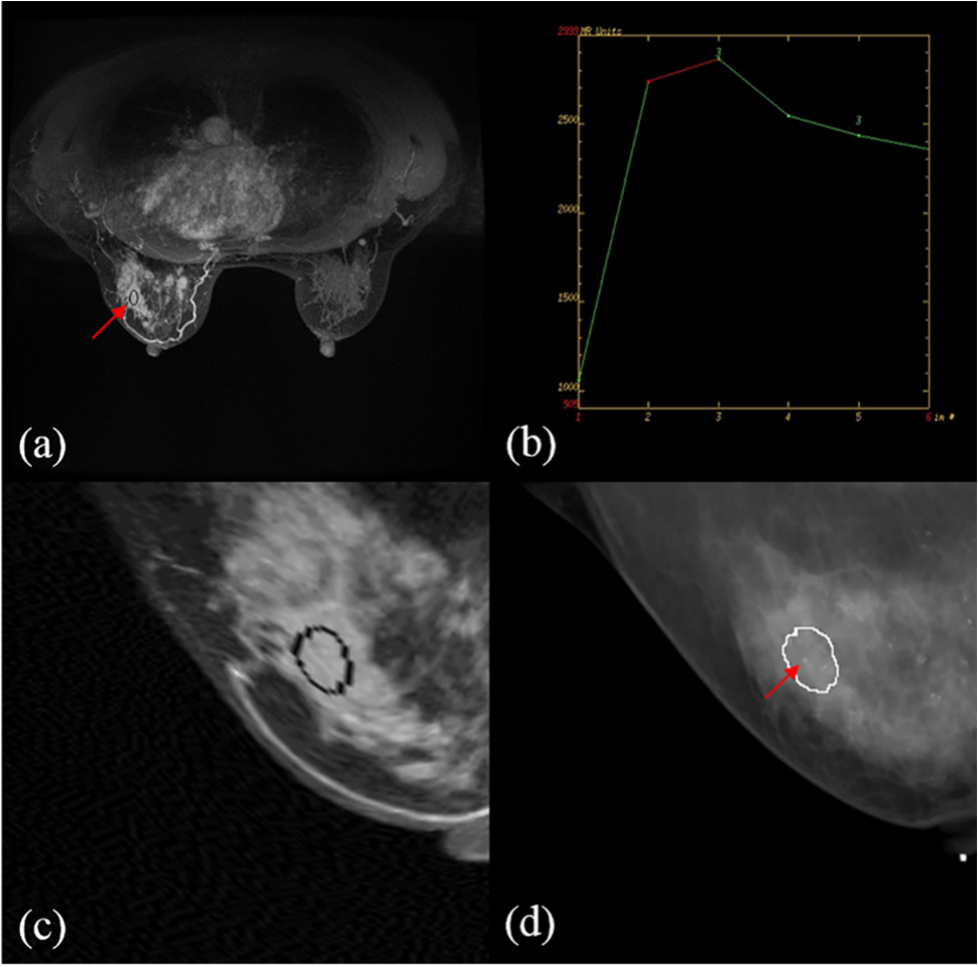
Illustration of the pre- and post- registration of breast MRI with annotation to fuse on mammography in a selected patient for the CC view. (a) the annotation (red) in MIP of MRI (b) (e) the annotation in mammography (green) (c) the fused annotations from MRI to the mammography (d) the registered MRI with annotation (red) (f) the fusion of registered annotation from MRI to mammography. The yellow area is the overlapping part between the annotations of the fused MRI and mammography.

**Fig 4:**
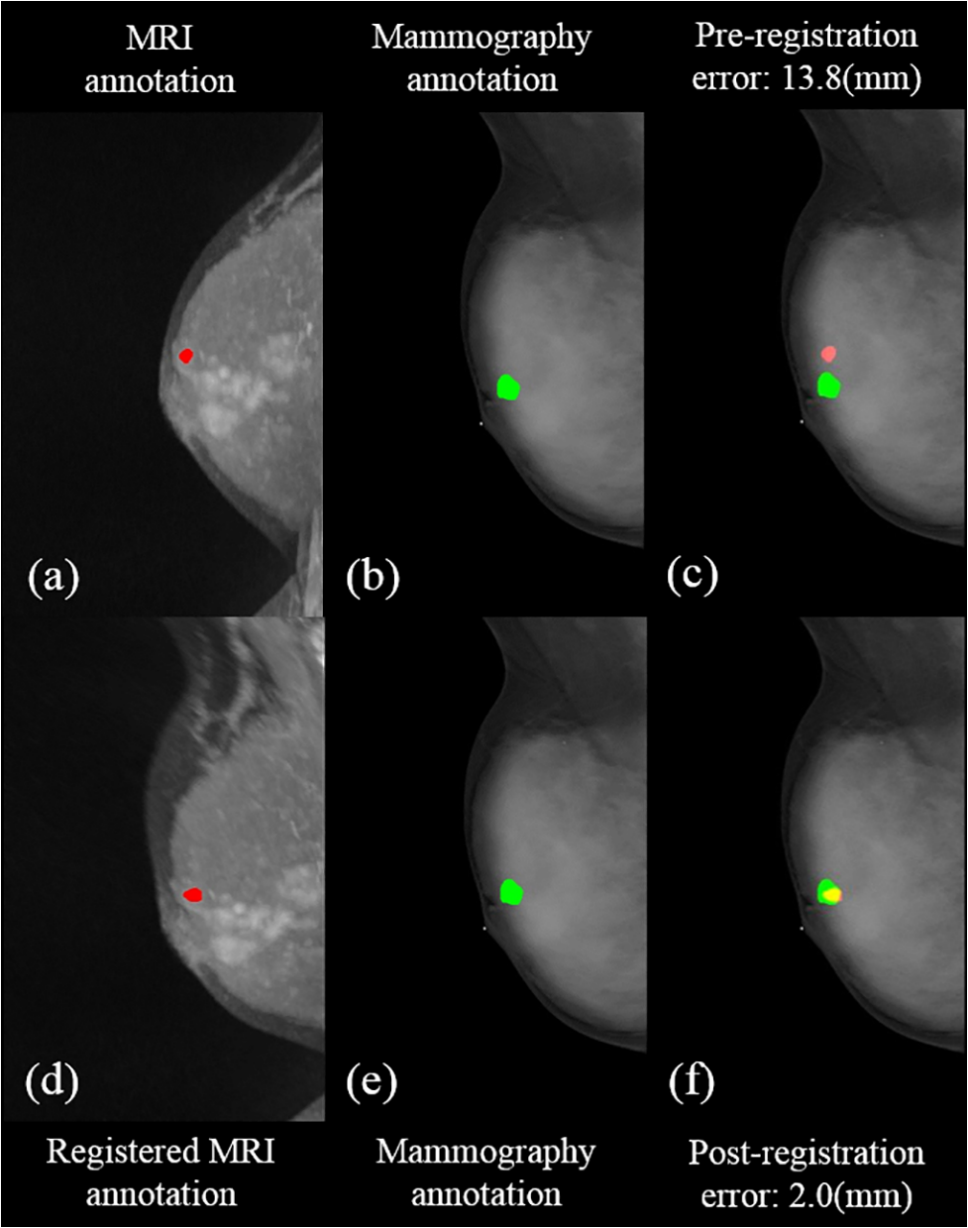
Illustration of the pre- and post- registration of breast MRI with annotation to fuse on mammography in a selected patient for the MLO view. (a) the annotation (red) in MIP of MRI (b)(e) the annotation in mammography (green) (c) the fused annotations from MRI to the mammography (d) the registered MRI with annotation (red) (f) the fusion of registered annotation from MRI to mammography. The yellow area is the overlapping part between the annotations of the fused MRI and mammography.

**Fig 5:**
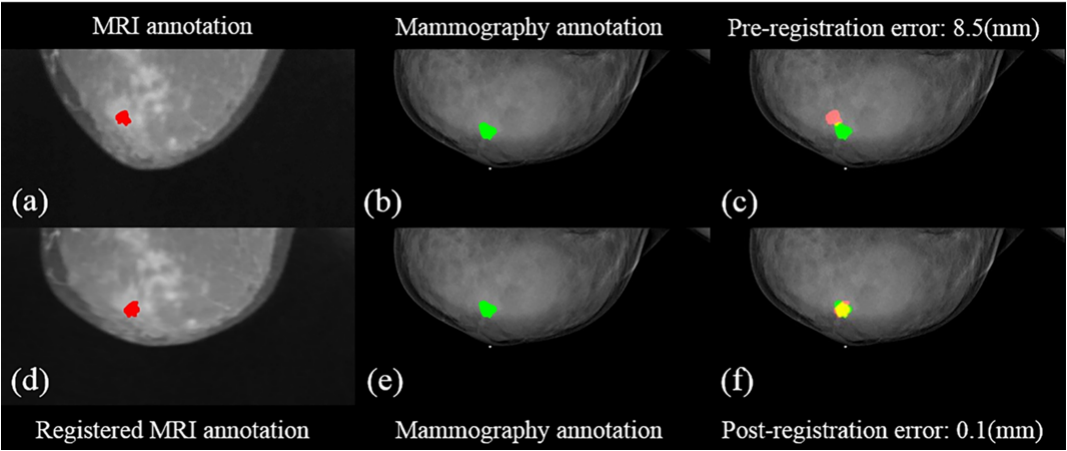
A 54-year-old woman with invasive ductal carcinoma in the upper outer quadrant of the left breast. (a) MRI annotation (b) the kinetic curve for MRI annotations indicating the Type 3 for the lesion (c) the deformed MRI annotation to register the mammography (d) the microcalcification observed in mammography in corresponding contour.

The errors before and after the registration with the assigned control points are shown in Table 3. For the CC view, the mean post-registration error between MRI and mammography was 2.2±1.9 mm, which was better than that of pre-registration (14.9±6.6 mm). For the MLO view, the proposed approach performed even better with a mean error of 3.0±2.4 mm, compared to 24.5±12.4 mm of the pre-registration. Figs 3 and 4 illustrate a representing example with the pre- and post- registration of breast MRI with lesion fused on mammography for the CC view and MLO view, respectively. An example showing the diagnosis of delineation by integrated information from MRI and mammography is presented in Fig 5. The comparison of histological results with indication of MRI assessment with/without information of mammography is shown in Table 4. The results by MRI alone were: the sensitivity 81% (25/25+6), specificity 53% (8/7+8), accuracy 72% (25+8/46), PPV 78% (25/25+7) and NPV 57% (8/6+8). In the diagnosis using MRI assessment with information of mammography, they were sensitivity 91.9±2.3%, specificity 70.0±4.7%, accuracy 84.8±3.1%, PPV 86.4±2.1% and NPV 80.8±5.4%. The kappa value (κ = 0.718) indicated substantial agreement of diagnostic performance of the radiologists.

**Table 3:** The errors of pre- and post-registration with the assigned control points for CC and MLO view.

View	Control point	Error (mm)	
		pre-registration	post-registration
CC	8–13	14.9±6.6	2.2±1.9
MLO	9–14	24.5±12.4	3.0±2.4

**Table 4:** The comparison of histological results with indication of MRI assessment with/without information of mammography.

	Diagnosis	
Breast MRI	Malignancy	Benign
Positive	25	7
Negative	6	8
Radiologist#1		
Microcalcification (+)	29	4
Microcalcification (-)	2	11
Radiologist#2		
Microcalcification (+)	28	5
Microcalcification (-)	3	10

## Discussion

Previous studies have demonstrated that MRI alone is rather sensitive than specific as an imaging method for detecting breast carcinoma [10–13]. The present study shows the similar results with 81% of the sensitivity and 53% of specificity in our patient cohort. However, the limited in specificity while using MRI alone for diagnosis of breast cancer can be improved by the combined information of MRI and mammography as the proposed approach. The specificity was significant increased from 53% to 70% in our study (Table 4). The sensitivity, accuracy, PPV and NPV were also improved for the evaluation as shown in Table 4. Non-mass lesions were the major cause of false-positive breast MRI findings [26]. The 3 of 7 cases of false-positive diagnosis for non-mass lesions in MRI scans were observe in our received data. With information combination of MRI and microcalcification distribution for further evaluation, more accurate diagnosis of breast cancer, especially for non-mass enhanced lesions, can be reached, as shown in Table 4.

Jiang et al. reported useful diagnosis of breast cancer with evaluation by integration MRI and microcalcification information from mammography [27]. They selected homologous regions of MIP image compared with microcalcification regions of mammogram for gathering the information from two modalities. However, the image registration for corresponding areas between MRI and mammography was absent in the study. Consequently, identifying corresponding regions from one image to the other cannot be confirmed with validation. In fact, studies have investigated to mitigate MRI to X-ray registration and the finite element method (FEM) based approach is one of the growing research areas. Hopp et al. spatially matched the MRI data with the mammography images applied by FEM and showed improved lesion registration [21], with the cost of increasing complex in biomechanical models, and the registration accuracy is determined by a number of patient-specific parameters. Another study including the rigid-body transformation on FEM was also reported by Mertzanidou et al [22]. These newest studies reported the accuracy of registration from MRI to mammography is approximate 10 mm. Our results show that the MIP MRI incorporated of TPS works well for the registration of annotation from MRI to both CC and MLO views of mammography with a better accuracy of 2–3 mm. The larger errors of registration in MLO view than in CC view were observed in our results. The mammography in MLO view is the projection image generated from 45° angle. The variation of set-up angle by different technologists may be the cause which may affect the subsequent MLO view generation and account into the accuracy of registration.

One major concern in this study is that the image registration was done by manual selection of control points for TPS, which requires familiarity of anatomy. Thus, the present approach for the radiologists and the technologists who are not professional in human anatomy is not a possible option. In general, the performance of registration with the control points selection and program execution is within 20 minutes for a single case, including the generation of MRI to CC and MLO views and registration to mammography.

In this study, we have developed a framework of registration from breast MRI to mammography and evaluated the clinical diagnosis of breast tumors on patients using integrated information from MRI and mammography. Breast cancer diagnosis using MRI with the aid of information from mammography shows potential improvements of sensitivity, specificity, accuracy, PPV and NPV as compared to the diagnosis of MRI alone.

Currently, there are many new technologies that offer better diagnostic accuracy than the conventional mammography, such as digital mammography, tomosynthesis, etc. MRI, as an additional imaging method together with the framework we present in this paper, can also provide better diagnostic results with those better technologies. Registration with determining corresponding regions between an MRI and a new complementary modality is in our future research plan.
